# Environmental Factors in Birth Defects: What We Need to Know

**Published:** 2009-10

**Authors:** Bob Weinhold

**Affiliations:** **Bob Weinhold**, MA, has covered environmental health issues for numerous outlets since 1996. He is a member of the Society of Environmental Journalists

Given the myriad steps involved in fetal growth, each presenting the opportunity for developmental mischief, it is not surprising that more than 7,000 kinds of birth defects are known to occur. The causes of birth defects remain largely a mystery, however, although a few culprits have been identified as important contributors, including some environmental agents.

In the developing world, where malnutrition, poverty, disease, and lack of access to health care elevate prenatal risk, birth defects impose “enormous personal and societal consequences,” according to the 2003 Institute of Medicine report *Reducing Birth Defects: Meeting the Challenge in the Developing World*. Experts in the developed world often have similar concerns, because in many regions birth defects are the largest single cause of infant deaths.

But reliable numbers for both defects and cases remain unclear, due in large part to a lack of funding, coordinated monitoring efforts, and adequate data. The causes of only about 30% of birth defects are somewhat well understood, and knowledge even of those is sometimes spotty. The 70% still unknown leaves open the possibility that environmental factors could play a significant role. “There are all kinds of classification and data challenges that are tough to overcome,” says Ted Schettler, science director of the nonprofit Science and Environmental Health Network. “It almost makes cancer tracking look easy—[those researchers are] getting pretty standardized data that’s completely not available in the world of birth defects.”

## Defining “Defects”

There is no universally accepted definition of what constitutes a birth defect. The term is sometimes limited to apparent structural problems, but often is expanded to include defects in function, metabolism, or body chemistry that lead to physical or mental problems or to death. Premature birth and low birth weight, which are linked with numerous health problems later in life, also can be influenced by birth defects, although factors such as young maternal age and poor access to medical care contribute as well. Birth defects also can be a contributing factor to some miscarriages and stillbirths.

Worldwide, at least 7.9 million people are born each year with a birth defect, according to *Global Report on Birth Defects: The Hidden Toll of Dying and Disabled Children*, a 2006 report by the March of Dimes Foundation, a birth defects research and advocacy group. The authors say their report is the first to provide global, country-by-country data on birth defects, with information from more than 190 nations. The report is especially helpful as a springboard for further investigation because 85% of the world’s birth defects occur in developing countries, but only four such countries have birth defect programs that can track occurrences, says Li Zhu, director of China’s National Reference Laboratory on Reproductive and Child Health.

Of the children affected by birth defects, at least 3.3 million worldwide die each year before age 5, according to the *Global Report*. Of those that survive, about 3.2 million will be mentally or physically disabled for life, although some of these effects may be mitigated with appropriate care. Actual numbers may be much higher, according to some experts, because they don’t account for certain functional and developmental birth defects, and because surveillance data on birth defects remain relatively primitive. Moreover, elective abortion after defects are detected via screening can mask the true incidence.

Based on the report’s estimates, birth defects affect about 4% of live births in high-income countries and about 8% in low-income countries, with a global average of about 6%. In the United States, the Centers for Disease Control and Prevention (CDC) estimates 3% of children born each year have a major structural birth defect that appears by the sixth birthday. That translates to about 130,000 U.S. children per year, although other estimates put the number as high as 325,000. These and other estimates often don’t include stillborn deaths, of which about 16% are caused by birth defects, according to the California Birth Defects Monitoring Program.

Figures from the National Center for Health Statistics indicate that birth defects cause about 20% of infant deaths in the United States. There are large geographical differences in the United States for infant death rates overall and for deaths attributed to birth defects. For deaths associated with birth defects, which averaged 0.43% of all infant deaths from 1999 to 2005, the South and Midwest are both about 40% higher than the Northeast. These large geographic variations readily allow for the possibility that variations in environmental exposures may be playing a role. They also reflect differences in local culture, socioeconomics, health care, and genes (some of these factors can in turn influence the rate of elective abortion). But they likely also reflect reporting and data quality variations, says Craig Mason, president of the National Birth Defects Prevention Network—hence the need for improved surveillance systems at a time when such programs are being cut or risk being eliminated altogether.

Lorenzo Botto, an associate professor in the University of Utah Division of Medical Genetics, agrees, saying, “A real problem is lack of funding. Birth defect programs are being cut, even in terms of basic surveillance infrastructure, or funded just to maintain basic monitoring capacity. This is true in the United States [but] even more so in developing countries, where there is not much spare capacity to look at environmental studies.”

## Environmental Red Flags

When it comes to fetal exposures, any exposure that occurs by way of the mother typically is considered “environmental.” A relatively small proportion of birth defects can be attributed, at least in part, to specific environmental causes such as maternal disease (e.g., rubella) or use of pharmaceuticals (e.g., valproic acid, an anticonvulsant and mood stabilizer). However, the majority of birth defects are considered the result of multiple environmental and/or genetic causes acting together. Lack of sufficient folic acid in the diet, for example, is one environmental factor now being remedied with supplements and food fortification. Among other environmental exposures with some incriminating evidence are other nutrient imbalances, maternal smoking and alcohol use, pesticides, tap water disinfection by-products, plastics and plastics components, solvents, metals, and numerous air pollutants (see table, p. A446).

Pinning down the role of environ mental agents remains a daunting challenge, however. “Of all the antecedents of birth defects, environmental factors are least well understood,” says Michael Katz, March of Dimes senior vice president for research and global programs. “This is inevitable inasmuch as there are few isolated influences. In most situations the environment imposes an avalanche of possible stimuli, some beneficial, many neutral, and others possibly harmful. Add to this the possibility that interactions between two such factors—or more likely among many—may result in different effects, and you have an inkling of the complexity of the problem.” Officials from the California Birth Defects Monitoring Program also point to the difficulty of extrapolating from animal data to human health effects, our lack of knowledge about the role of genetic factors in birth defects, and the lack of data on both exposures during pregnancy and the effects of specific exposures on the fetus.

Many experts are concluding that genetic factors are always implicated in birth defects. “We don’t know of any environmental exposures where a hundred percent of kids are affected,” says Tina Chambers, a perinatal epidemiologist in the Division of Dysmorphology and Teratology at the University of California, San Diego. “We think there are gene–environment interactions with all these [cases].” However, even with the many environmental red flags that have been raised so far, the field of investigation remains vast. “We really don’t know the right questions to ask,” Chambers says.

## Crawling Toward a New Path

There’s no shortage of ideas on ways to improve our understanding of birth defects and the role of the environment. Stuart Newman, a professor of cell biology and anatomy at New York Medical College, is in the camp of those recommending major shifts in thinking. He would like to see birth defects as a whole addressed at multiple levels from the micro to the macro. “It will take a more integrated and holistic approach,” he says. His preference is to meld the fields of genetics, developmental biology, evolutionary developmental biology, environmental science, and sociology—fields that often have a near or total disconnect among them—although he acknowledges there aren’t yet good models for how to do this.

Betty Mekdeci, executive director of the advocacy group Birth Defect Research for Children, says there are many problems with the basics of how birth defects are tracked and evaluated. Her experience of more than 30 years—prompted by her efforts, and those of her husband, to figure out why their son was born with multiple birth defects—led her to conclude that some of the most important limitations include inadequate medical diagnostic codes for classifying many birth defects, inaccurate use of codes by health care practitioners to meet insurance billing requirements, and the inability of many health care practitioners to diagnose a birth defect at birth or in follow-up visits, and skepticism toward the input of parents, who usually know better than any one doctor about the full range of health problems their child is having.

To overcome some of these problems, Mekdeci and her colleagues have developed an alternative method of tracking birth defect incidence based on parent responses to a lengthy questionnaire. About 6,000 completed questionnaires have been collected since 1990. Mekdeci and her staff analyze the questionnaire responses for patterns, and she reports they have identified about half a dozen clusters so far. Although the group readily acknowledges these are self-reports from a self-selected population, some of the clusters have later been confirmed by various government agencies. For instance, in Dickson, Tennessee, they detected a cleft palate cluster that was confirmed by the CDC. The group sees its role as identifying birth defect cases and then encouraging health agencies to investigate.

Assuming the current modes of thinking and operating continue for a while, Schettler says better use of existing information and resources can still be very beneficial. “We should be doing some cross-agency work to better simulate [exposures in] the world we live in,” he says. “It’s sort of a failure of our [research] system to not think out of our silos.” As one example of such failures, he cites evidence based on animal studies that the teratogenic action of valproic acid is exacerbated with concurrent zinc deficiency and reduced with zinc supplementation. Because zinc deficiency affects an estimated 25–40% of the general population worldwide, according to a report by Wolfgang Maret and Harold H. Sandstead in the 10 May 2006 *Journal of Trace Elements in Medicine and Biology*, this interaction may be very important to public health, he says. However, it has rarely been studied in humans.

Put another way, says Helen Dolk, project leader for EUROCAT (European Surveillance of Congenital Anomalies), “On the one hand we act too little on the environmental causes we know about, and on the other hand we do too little research to find out more about the environmental causes.”

However, Dolk points out, “There is surveillance, and there is research, and there is a spectrum between the two. We need specifically designed research—this does not need to be nationwide, but components of it will need very big population numbers, as birth defects are relatively rare. Research will be largely hypothesis-testing. . . . We also need surveillance, or tracking, which will throw up areas of concern and is more frequently hypothesis-generating. Putting all our resources in one of these areas without the other will not achieve the results we need.”

Moreover, she adds, “Funding often follows ‘fashions’ and dogma, and we need a balanced approach, judging case–control, cohort, and other studies on their own merits, rather than completely turning from one type to another.”

## Priorities for Change

Even if none of the recommended shifts in overall approach occur, other major adjustments are needed before much more progress can occur, says Christopher Howson, vice president for global programs at the March of Dimes. Paramount among these is more leadership and guidance from the World Health Organization (WHO). “The WHO is a very important player in international health,” Howson says. “To get the imprimatur of the WHO gives health ministers the authority they need to address [specific health] issues.”

The WHO has addressed birth defects to some extent, but the issue “has not been on the priority list of the WHO agenda yet,” Zhu says. Dolk adds, “The WHO needs to incorporate prevention of birth defects into programs to tackle major health determinants and improve maternal and reproductive health, recognizing the importance of environmental causes of birth defects.”

Howson says it’s critical to have individual countries establish a consistent national policy and approach for certain key elements, including the birth defects covered and the identification and tracking methods used. Within such a framework, he recommends designating a point person in one federal agency in each country as having central responsibility. That official would coordinate with representatives from other agencies to orchestrate consistency of identification, tracking, and reporting methods; agreement on what birth defects to track; and even basic logistical elements such as ensuring that software packages used are compatible with various computer systems. With that kind of structure in place, he would like to see all health-related programs in a country incorporate relevant birth defect information into their work, whether it’s related to nutrition, disease, pollutants, genetics, or other fields. He also would like to see the WHO and individual countries develop extensive education programs to inform both citizens and health professionals about birth defects, known causes and remedies, and other basic information.

Dolk raises another issue—that of data confidentiality and parental consent for babies with birth defects to be included in surveillance systems. “Experience shows that most parents do not refuse [to participate] and even expect this for the good of future babies,” she says. “However, requirements to obtain consent can be logistically very difficult and costly to implement—in a number of European countries this problem has not been solved adequately to allow data to be fully used.”

In still another arena, experts are split to some degree on whether a centralized national health system, in which everyone’s comprehensive health records are accessible from one source, is necessary for robust surveillance. Dolk says, “Experience has generally been that centralized national systems result in poor data compared to decentralized systems closer to those producing and using the data.” However, she adds, this depends on the size of the country and on the types of information systems available for use and linkage. “Thus,” she says, “national Scandinavian systems work well.”

Norway and Denmark have extensive data for just about everyone in the country, including health records, socioeconomic and occupational information, and other demographics. “These countries have been able to do some very cool linkage studies,” says Andrew Olshan, chairman of the Department of Epidemiology at the University of North Carolina at Chapel Hill. Some of those studies have been conducted through the Norwegian Mother and Child Cohort Study and the Danish National Birth Cohort Study. Each study is evaluating about 100,000 women and their children over the course of at least 15–20 years for a wide range of factors, including selected birth defects. However, evaluation of any links between birth defects and environmental exposures is likely to be limited due to constraints on the quality and availability of data for specific toxic exposures.

Margaret Honein, Birth Defects Branch chief in the CDC’s National Center on Birth Defects and Developmental Disabilities, believes a decentralized approach also can work. “I think what’s needed is to make sure it’s possible to do good surveillance, with nothing obstructing [state programs],” she says. “What’s most important is to get high-quality data from all the programs.” However, in the United States, about 10% of states don’t even have a birth defects registry, about 20% have very limited data, and the remaining 70% have data that often can’t be compared state-to-state, according to “Population-Based Birth Defects Surveillance Data from Selected States, 2001–2005,” a CDC report that constitutes the December 2008 issue of *Birth Defects Research: Part A: Clinical and Molecular Teratology*.

Even more limiting, from an environmental perspective, is that two-thirds of U.S. states don’t explore any links between birth defects and environmental exposures, according to a 2005 report from the health advocacy group Trust for America’s Health titled *Birth Defects and Developmental Disabilities: The Search for Causes and Cures*. The number of states that track such links in at least some way may be slightly higher now, says Mason, although he reiterates that recent economic problems are causing some states to cut back on their birth defect programs.

## Time for a Parade?

The National Children’s Study (NCS), just getting under way in the United States, is expected to include extensive data on the effect of environmental exposures on fetal and child health. The NCS offers the best chance in the near term of improving our knowledge of links between environmental factors and birth defects, according to Chambers.

Most birth defects research to date has been conducted retrospectively, relying on recall of study participants of events often occurring many years earlier, and lacking data on actual specific exposures of participants. In contrast, the NCS aims to enroll 100,000 women from representative populations and follow their children from conception through age 21. The NCS will collect biological samples from parents and children as well as samples of air, drinking water, interior dust, and soils from participants’ homes. These will be stored along with all medical records, giving future researchers the ability to evaluate a wide range of structural, functional, and developmental birth defects, says NCS acting director Steven Hirschfeld—even though we may not have the insights or tools to know what to look for or how to look for it today.

Chambers, who is a principal investigator in the study, says early results should begin to trickle out in about five years; data on birth defects that show up soon after birth will be among the first available for each participant.

Even more statistical power could someday be available by combining study resources. A prototype for such research is the International Childhood Cancer Cohort Consortium (I4C), which is just beginning to analyze its first batches of data. The I4C project is an ambitious effort to facilitate apples-to-apples comparisons among multiple studies for specific end points. The initial focus is on childhood leukemia, which has been linked for many years with Down syndrome. In a review published in the February 2009 issue of *The Oncologist*, Karen R. Rabin and James A. Whitlock note that children with Down syndrome have a 10- to 20-fold higher risk of some form of childhood leukemia, although studies of potential environmental factors connecting the two conditions have been inconclusive. However, recent research is showing that the relationship between childhood leukemia and genetic abnormalities present at birth may extend further, says Carol Kasten, a geneticist at the U.S. National Cancer Institute—and I4C is expected to provide more insights.

The initial I4C group, whose first workshop was held in 2005, included 11 cohorts from 9 countries. Some of these studies were already well under way, others only just starting. Among those with higher numbers of participants are the NCS in the United States, the Danish and Norwegian studies, and studies in China and Israel. Others have far fewer participants, sometimes just a few thousand. But all combined, I4C includes about 700,000 mothers, and at least some of the cohorts will be able to provide data on environmental factors such as pesticides, tobacco smoke, and electromagnetic fields.

Research projects from other countries that have been invited to attend a November 2009 I4C conference in Lyon, France, may also join the group. These include projects in Brazil, Canada, Japan, the European Union, South Korea, and South Africa. A second Chinese program (in addition to the study already participating in I4C) currently in its pilot phase could add hundreds of thousands more mothers.

If the I4C program works as expected, it could bode well for birth defects research. “I’m sure we’ll get to birth defects at some point with I4C,” Kasten says. A similar program focusing specifically on birth defects of all types doesn’t exist yet, Kasten says, “but certainly people are wondering, ‘Why don’t we do that?’”

In addition to these efforts, a few studies of links between certain birth defects and environmental agents have been conducted using data from some of the 43 registries in 20 countries that participate in EURO-CAT. This network of European population-based registries began in 1979 and today includes about 30% of the European birth population. A parallel compilation of continent-wide data has occurred since 1974 through the Latin American Collaborative Study of Congenital Malformations. This study focuses on genetics, pharmaceuticals, alcohol, smoking, nutrition, and maternal disease, but links to pollutants are studied extremely rarely. The International Clearinghouse for Birth Defects Surveillance and Research, also operating since 1974, provides a forum for collaboration among countries from around the world. It does not itself offer a specific mechanism for tracking impacts of specific environmental agents, but some of its members do conduct such research.

Still more information on environmental links to birth defects may come from the Metropolitan Atlanta Congenital Defects Program, one of the oldest such programs in the world, operating since 1967. It is limited in geographic coverage, including residents of just five central-Atlanta counties, but it conducts active surveillance of major structural birth defects through doctors’ offices, clinics, and genetic laboratories rather than relying on hospital birth records, which is a limitation of some registries. Whereas many registries stop at birth or after the first year of life, the Atlanta program follows children up to their sixth birthday, which is important because some defects are not associated with any symptoms until the child is older. Honein says a few projects involving environmental substances (which she declined to name) are in the early stages, and results from initial studies may be available within a year or so.

In addition to the Atlanta program, four of the nine CDC-supported Centers for Birth Defects Research and Prevention may soon begin to publish a few insights related to various environmental exposures, Honein says. These centers are participating in the National Birth Defects Prevention Study, the largest etiologic study of its type ever conducted. Each year, each center identifies approximately 300 new mothers of children with common birth defects as well as 100 control mothers. Doctors and researchers review each case in detail, looking for known or suspected factors. Various centers are studying agricultural pesticides, disinfection by-products, and hazardous waste emissions, among other exposures. The study began in 1997, and the database now includes about 25,000 births. In addition, CDC officials are in the early stages of considering studies of additional air, water, and soil pollutants and utilizing other environmental data, Honein says.

Other efforts that may generate information related to environmental exposures are expected to trickle out via other independent and government agency research around the world. But although these efforts to improve information about environmental and other causes of birth defects offer some potential for expanded knowledge, the broader-scale limitations of making significant advances, including a perennial lack of funding, leave some critics skeptical.

Others are more optimistic, however. Howson sees the progress made so far, and the groundwork that has been laid for future progress, as something akin to a favorite children’s activity: “It’s like a kid going out into the street to bang his drum. Another kid joins him, then another and another. By the end of the street, you have a parade.”

## Figures and Tables

**Figure:**
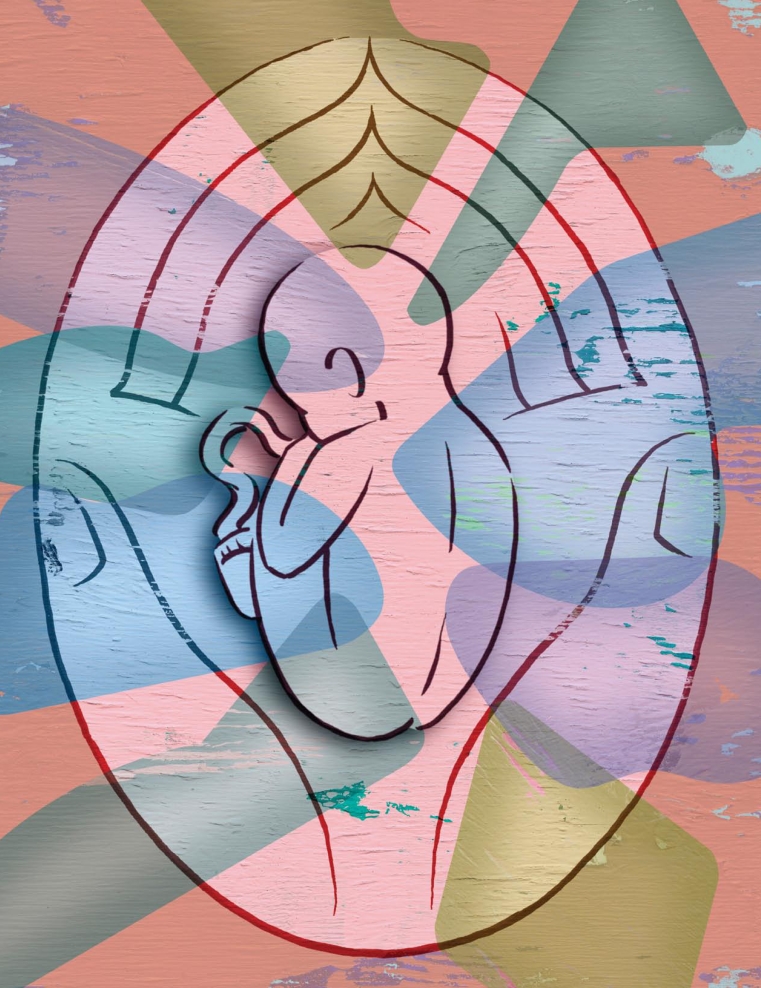
Worldwide, at least 7.9 million people are born each year with a birth defect. Of the children affected by birth defects, at least 3.3 million die each year before age 5, and about 3.2 million of surviving children could be mentally or physically disabled for life. Currently the causes of only about 30% of birth defects are even somewhat well understood. —Global Report on Birth Defects: The Hidden Toll of Dying and Disabled Children

**Figure:**
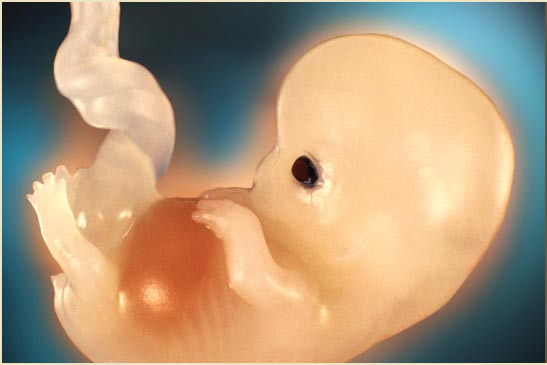
There are all kinds of classification and data challenges that are tough to overcome [in birth defect surveillance]. It almost makes cancer tracking look easy. —Ted Schettler, Science and Environmental Health Network

**Figure:**
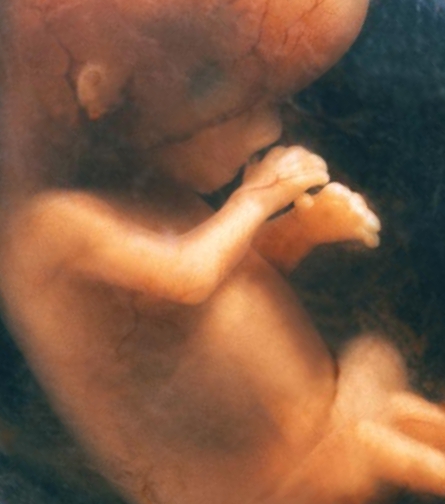
In most situations the environment imposes an avalanche of possible stimuli, some beneficial, many neutral, and others possibly harmful. Add to this the possibility that interactions between two such factors— or more likely among many— may result in different effects, and you have an inkling of the complexity of the problem. —Michael Katz, March of Dimes Foundation

**Figure:**
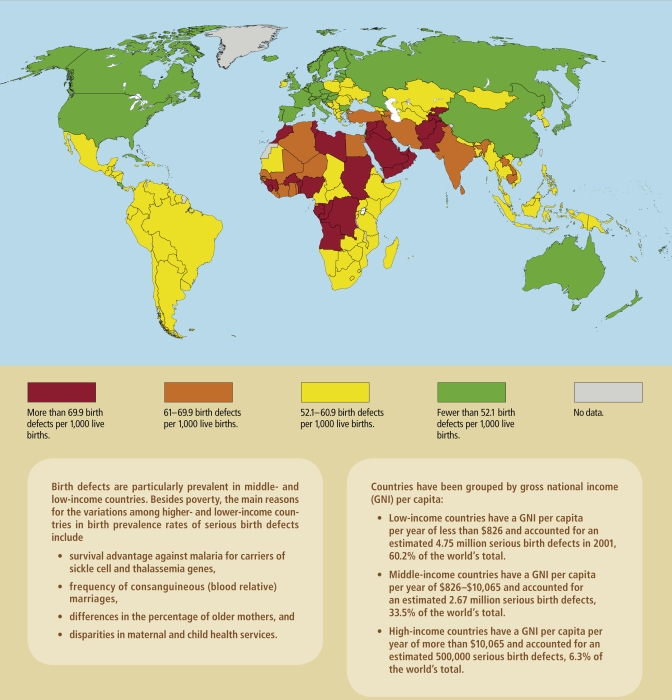
Birth Defect Rates Around the World, 2001 **Source:** March of Dimes Foundation

**Figure:**
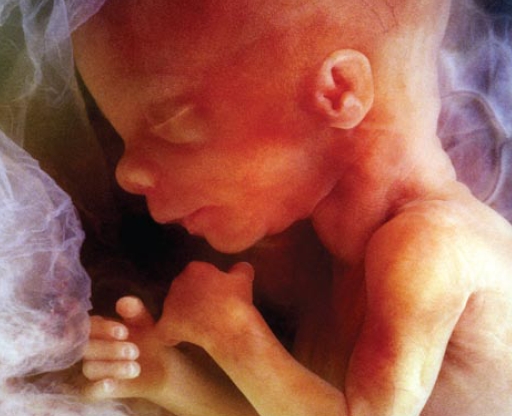
There is surveillance, and there is research, and there is a spectrum between the two. . . . Putting all our resources in one of these areas without the other will not achieve the results we need. —Helen Dolk European Surveillance of Congenital Anomalies

**Table:** A Sampling of Environmental Agents Studied in Relation to Birth Defects This list is not a comprehensive catalog of all environmental agents studied in relation to birth defects, nor should it be construed to mean that all the agents listed below have been established as risk factors for birth defects. The strength of evidence varies widely for each of the agent/birth defect associations listed below. Associations were studied in humans except where noted.

Agent^1^,^2^	Birth Defect(s)
Accutane^3^	Head, facial, cardiac, neurologic, thymus defects
Alcohol	Cardiac defects, oral clefts, fetal alcohol syndrome, cryptorchidism
Arsenic	Cardiac defects
Benzene	Cardiac, neural tube defects
Bisphenol A	Female reproductive defects
Botulinum type A toxin^3^	Hearing, vision defects
Brominated flame retardants	Genitourinary defects
Caffeine	Cardiovascular defects, altered protein function, increased body fat (mice)
Carbon monoxide	Cardiac, neurologic defects (rats)
Chlorophenoxy herbicides	Cardiovascular, musculoskeletal, respiratory, skin defects
Chlorpyrifos	Cardiac, facial, genitourinary, musculoskeletal, neurologic defects
Diclofop-methyl	Hypospadias
Diethylstilbestrol^3^	Reproductive defects, cryptorchidism
Dioxins	Neural tube, neurobehavioral defects; hypospadias; oral clefts
Disinfection by-products in tap water	Cardiovascular, esophageal, neural tube, urogenital defects; oral clefts; gastroschisis
Ethinylestradiol^3^	Increased pain sensitivity (rats)
Fumonisin	Neural tube defects
Glyphosate	Neurobehavioral defects
Ionizing radiation	Cardiac, neurologic, neural tube, skeletal defects; gastroschisis
Lead	Cardiac, neural tube, neurobehavioral defects; oral clefts
Mercury	Neurologic, hearing, vision defects
Methotrexate^3^	Skeletal defects
Methoxychlor	Increased pain sensitivity (rats)
Organic solvents	Neural tube, cardiac, limb defects; oral clefts; gastroschisis; developmental disorders
Organochlorine pesticides	Cryptorchidism, hypospadias
Ozone	Cardiac defects, cleft lip with or without cleft palate
Particulate matter	Vascular defects (patent ductus arteriosus)
Polychlorinated biphenyls	Hearing defects (rats)
Perfluorooctane sulfonate	Cleft palate (mice)
Phosphine	Neurologic, neurobehavioral defects
Phthalates	Male reproductive defects
Polycyclic aromatic hydrocarbons	Neurodevelopmental defects
Secondhand tobacco smoke	Neurobehavioral, cardiac, limb, respiratory defects; oral clefts; hypospadias; gastroschisis
Sulfur dioxide	Musculoskeletal, cardiac defects (cattle)
Thalidomide^3^	Limb defects
Trichloroethylene	Neural tube defects, oral clefts
Valproic acid^3^	Neural tube defects

1 Several diseases or their agents have links with various birth defects, including influenza, diabetes mellitus, phenylketonuria, myotonic dystrophy, myasthenia gravis, syphilis, Coxsackie virus B, cytomegalovirus, rubella, toxoplasmosis, and varicella zoster virus.

2 High maternal temperature, whether caused by disease, sauna, or other factors, can cause birth defects.

3 Numerous other pharmaceutical agents have been studied in relation to various birth defects.

